# Using mechanical testing to assess the effect of lower-limb prosthetic socket texturing on longitudinal suspension

**DOI:** 10.1371/journal.pone.0237841

**Published:** 2020-08-19

**Authors:** Julia Quinlan, Jessica Yohay, Vasanth Subramanian, Brad Poziembo, Stefania Fatone

**Affiliations:** 1 Department of Physical Medicine and Rehabilitation, Northwestern University Prosthetics-Orthotics Center, Northwestern University Feinberg School of Medicine, Chicago, Illinois, United States of America; 2 Prosthetic Design Inc, Dayton, Ohio, United States of America; University of Illinois at Urbana-Champaign, UNITED STATES

## Abstract

To function effectively, a lower limb prosthetic socket must remain securely coupled to the residual limb during walking, running and other activities of daily living; this coupling is referred to as suspension. When this coupling is insufficient longitudinal pistoning of the socket relative to the residual limb occurs. Increasing friction of the socket/liner interface may improve socket suspension and textured sockets may be fabricated relatively easily with 3D printing. The aim of this study was to investigate longitudinal displacement of sockets with different types of textures under two suspension conditions: passive suction and active vacuum. In order to do this, we developed a mock residual limb and mechanical testing protocol. Prosthetic sockets, 14 textured sockets and an Original Squirt-Shape (OSS) Socket, were fabricated from polypropylene copolymer using the Squirt-Shape^™^ 3D Printer and compared to a smooth socket thermoformed from polypropylene copolymer. Sockets were mounted onto a dual durometer mock residual limb and subjected to four levels of distraction forces (100 N, 250 N, 500 N and 650 N) using a hydraulic material testing system. There was a statistically significant three-way interaction between suspension, force level and texture (p < 0.0005). Longitudinal displacements between textured and reference sockets, for all force levels and both suspension conditions, were significantly different (p < 0.0005). Using these newly developed mechanical testing protocols, it was demonstrated that texturing of polypropylene copolymer sockets fabricated using Squirt-Shape significantly decreased longitudinal displacements compared to a smooth socket. However, none of the novel textured sockets significantly reduced longitudinal displacement compared to the OSS socket under passive suction suspension.

## Introduction

The lower-limb prosthetic socket is responsible for the distribution of load bearing forces between the residual limb and prosthesis. The socket must also remain securely coupled to the residual limb during activities; this coupling is referred to as suspension. In today’s prostheses, suspension occurs primarily through some amount of friction, pressure and shear forces [1]. Successful suspension, or pistoning reduction [2–4], provides improved control of the prosthesis [3, 5] by minimizing relative longitudinal movement between the socket and residual limb. In this case, longitudinal movement is defined as movement along the long axis of the tibia. While pistoning may be reduced through diverse suspension mechanisms including elastomeric or gel liners with mechanical locking pins or lanyards, or passive or active suction, with varying effectiveness reported [6–11], it remains an ongoing clinical problem [3, 4, 12–15].

While prosthetic sockets have conventionally been smooth, surface texturing of prosthetic sockets can be easily fabricated using three-dimensional (3D) printing. Rolock [16] developed a fused deposition modeling system (called Squirt-Shape) that fabricated sockets from polypropylene, a material that is commonly used in prosthetic socket fabrication. Original Squirt-Shape (OSS) Sockets have horizontal striations due to the 3D printing process and have been worn successfully by persons with lower limb amputation [17–19].

It is possible that socket suspension may be improved by increased friction properties of the socket/liner/residual limb [20–22]. A previous study reported increased coefficient of static friction when texture was added to polypropylene socket samples but no improvements in the coefficient of kinetic friction [21]. This was attributed to the American Society for Testing and Materials (ASTM) [23] standard for coefficient of friction testing being unable to engage the material surfaces in the same way they would when the socket is compressed around the residual limb. Hence, it was suggested that more realistic test set-ups are needed to assess the effect of texturing on socket suspension [22, 24].

Mechanical testing offers a potentially valuable approach for initial assessment of the effect of prosthetic socket texturing on suspension. Previous studies have utilized mechanical testing as a platform for exploring effectiveness of active vacuum suspension [25, 26] and the strength of a mechanical suspension system [27]. However, while a standard (e.g. Organization for Standardization ISO 10328 [28]) exists for structural testing of lower limb prosthetic components, it does not include prosthetic sockets. Hence, investigators have modified the ISO 10328 standard [28] to perform static, cyclic and fatigue tests of sockets [29–33]. To perform mechanical testing sockets need to be secured in the mechanical testing apparatus via a mock residual limb, which may vary in shape and material composition dependent on the aim of testing. Hard materials have been used to construct mock residual limbs when sockets have been tested to failure [32], while soft and compliant materials have been used to better mimic the surface of the residual limb when predicting biomechanical behavior at the residuum/socket interface [33] or when assessing socket fit and suspension under vacuum [25]. Choosing an appropriate mock residual limb design for mechanical testing of suspension forces is challenging. There is a need to transfer force effectively from the mechanical testing system through the mock limb to the socket interface while also having the compliance necessary to engage the textured surfaces. Hence, we developed a mock residual limb and mechanical testing protocols to investigate longitudinal displacement of sockets.

Since textures can be 3D printed to vary in pattern, depth and distribution, it is unknown what texture pattern might minimize longitudinal displacement. The aim of this study was to investigate longitudinal displacement of sockets with different types of textures under two suspension conditions: passive suction and active vacuum. We hypothesized (1) that novel textures would reduce longitudinal displacements compared to the smooth and OSS socket; and (2) that active vacuum would result in smaller longitudinal displacement for a given force compared to passive suction. Additionally, we considered whether more aggressive texture patterns and/or those with primarily horizontal pattern orientation, as compared to more subtle texture patterns and/or those with primarily longitudinal pattern orientation, resulted in less longitudinal displacement for a given force.

## Materials and methods

### Socket fabrication

**Fig 1:**
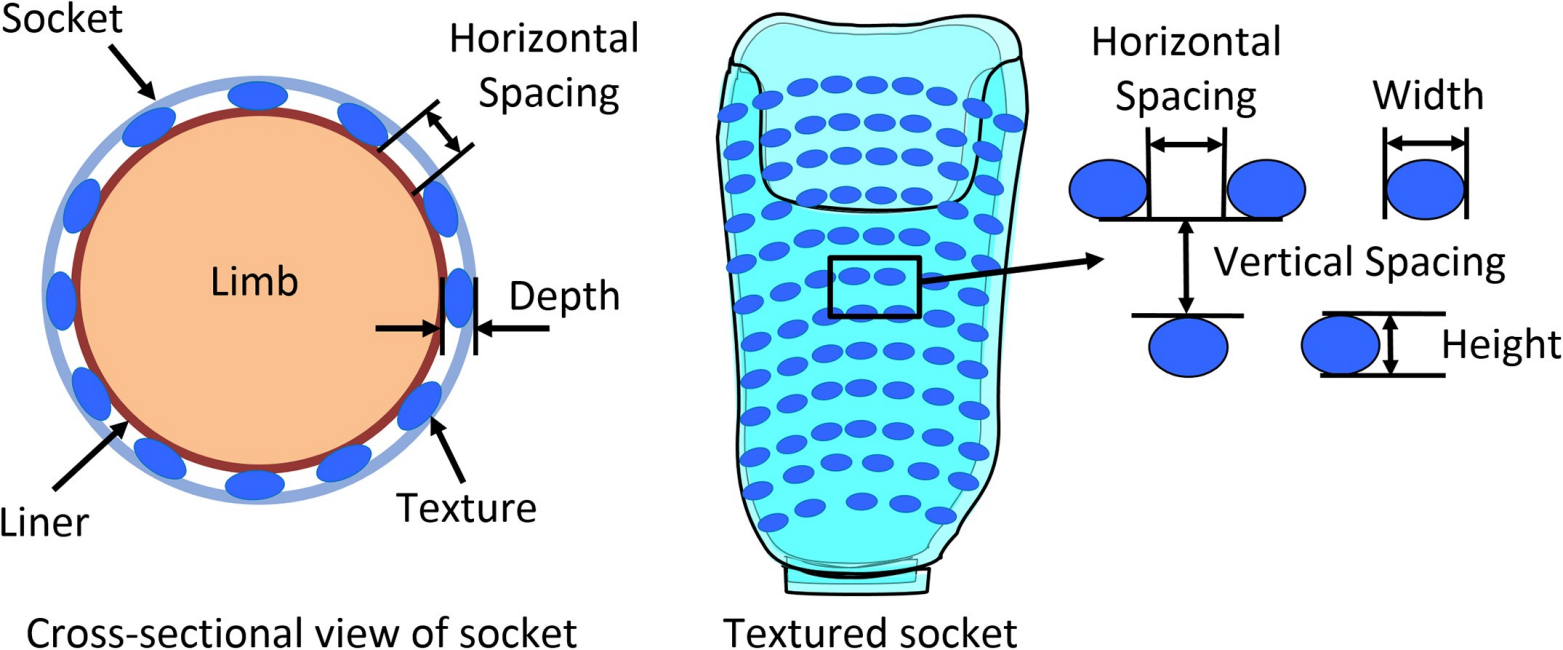
A. Cross-sectional and B. interior schematics of 3D printed prosthetic sockets defining texture dimensions.

**Fig 2:**
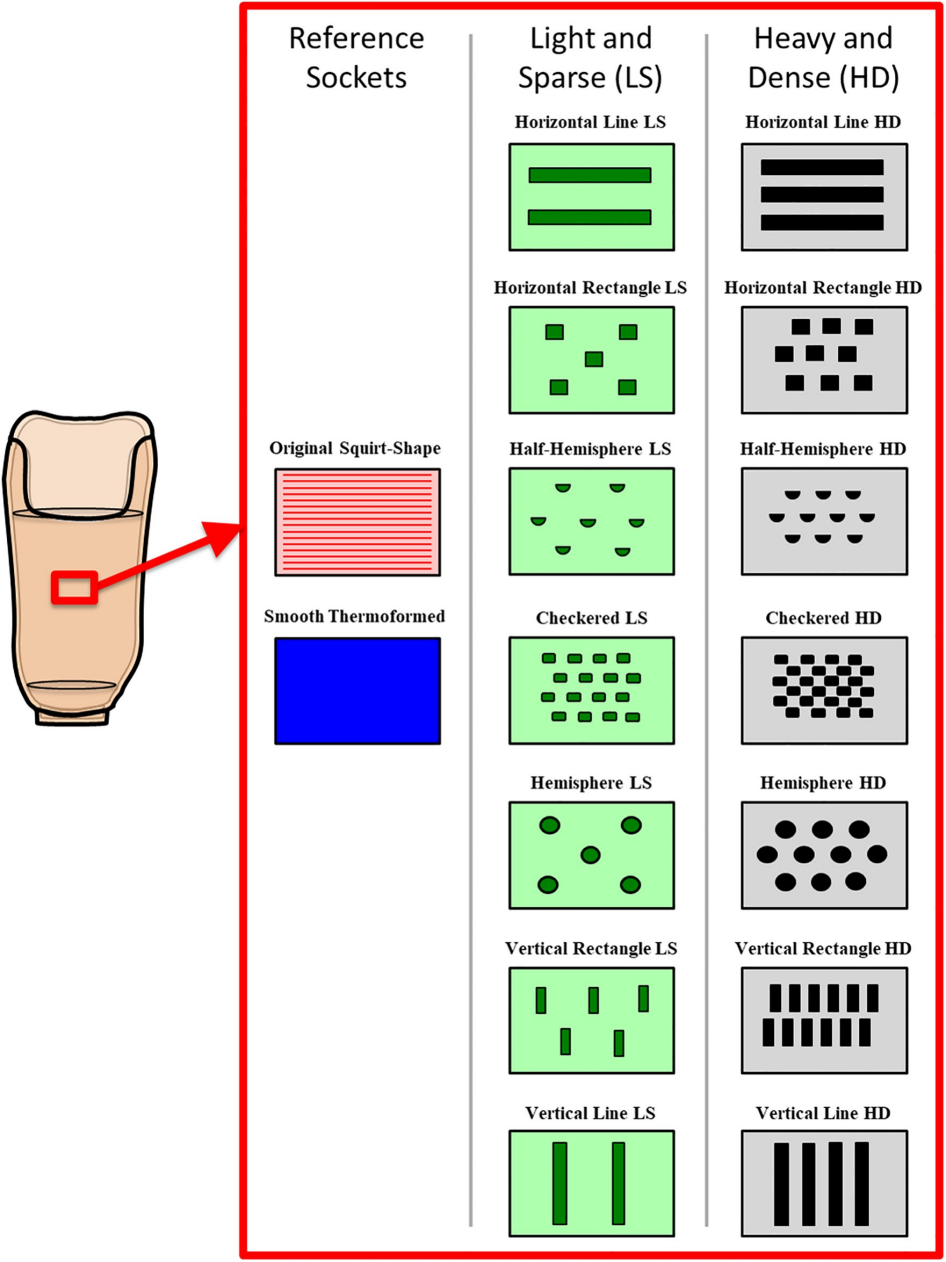
Schematic of the 14 novel texture patterns and 2 reference patterns assessed in this study. Textures are organized, top left to bottom right, from those with a primarily horizontal orientation of texture pattern to those with a primarily vertical orientation of texture pattern.

Prosthetic sockets with various combinations of texturing were fabricated using the Squirt-Shape^™^ 3D Printer (Prosthetic Design Inc., Dayton, OH) and polypropylene copolymer pellets (PPM-50, Prosthetic Design Inc., Dayton, OH) (Fig 1). Fourteen novel texture patterns were identified that could be successfully programmed and printed, including checkered, hemisphere, half-hemisphere, horizontal and vertical rectangles, as well as horizontal and vertical lines [22, 24]. Two sockets were printed for each of the seven texture patterns (Fig 2, S1 Appendix): “light and sparse” (LS, describing texturing with a smaller depth, where depth is the degree to which the texture protrudes into the socket volume, and sparsely distributed) and “heavy and dense” (HD, describing texturing with greater depth and more dense distribution) [22, 24].

For reference against our novel texture patterns, an OSS socket was fabricated using the same polypropylene copolymer pellets and having horizontal striations approximately 1.2 mm in depth and 0.75 mm in thickness [16, 17]. As a second reference, we also fabricated a smooth polypropylene socket (Copoly Natural White, North Sea Plastic, Endolite, Miamisburg, OH) using standard clinical thermoforming techniques. All sockets were fabricated to fit a mock transtibial residual limb covered with a 3 mm silicone prosthetic liner (Evolution Standard Liner, EOC-4, Össur Americas, Foothill Ranch, CA) and a nylon stocking.

### Mock residual limb fabrication

This study required fabrication of a mock residual limb capable of sustaining high testing loads, transferring loads reliably to the socket interface, and providing a compliant surface with which the socket textures could engage. To satisfy these criteria a dual durometer mock residual limb was fabricated having an inner, harder core (mimicking bone) and an outer, more compliant shell (mimicking muscle and skin). Materials that mimic skin and subcutaneous tissues have a shore hardness between 00A and 30A [34, 35], muscles have a shore hardness between 10A and 50A [35] and cortical and cancellous bone have a shore hardness between 65A and 85A [36, 37]. Hence, we fabricated a dual durometer mock residual limb using urethane rubber with shore hardness within these ranges.

A transtibial residual limb model was cast using plaster of Paris bandages to create the mold for the outer shell. To minimize adherence with the urethane rubber, the interior of the cast was sealed with two coats of one step sealer (One Step^™^, Smooth-On, Reynolds Advanced Materials, Chicago, IL) applied 20 minutes apart. Since the mock residual limb needed to withstand high mechanical loads, the thickness of the compliant shell was kept relatively small. Hence, a mold for the inner core was 3D printed using polypropylene copolymer (PPM-50, Prosthetic Design Inc., Dayton, OH) to create a shape approximately 2 cm smaller in circumference that fit inside the plaster cast. Plastic was used to fabricate the inner core mold as it did not bond to the urethane used for the outer shell and could be removed easily. The inner core mold was filled with liquid plaster and a mandrel centered inside.

**Fig 3:**
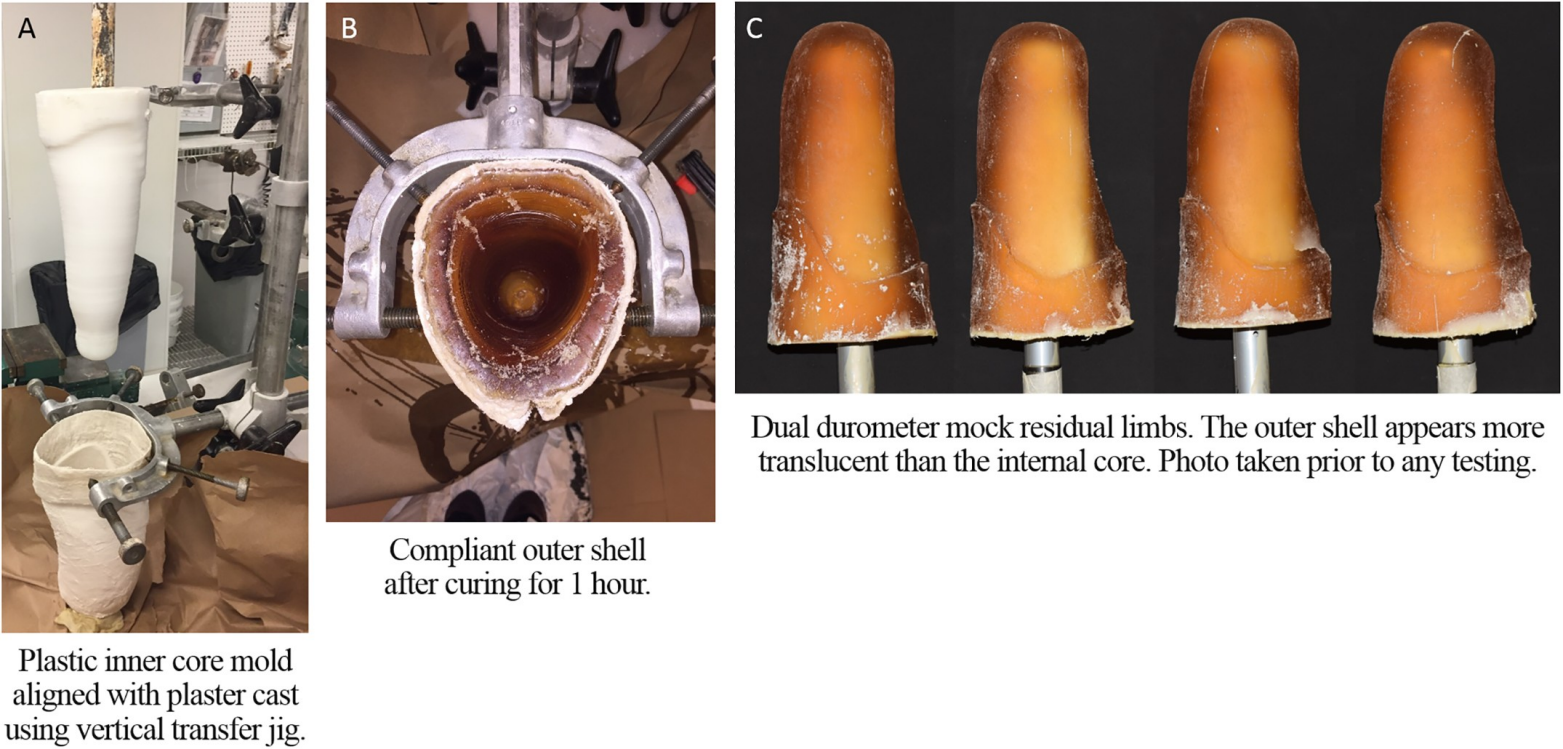
Fabrication of dual durometer mock residual limbs. A. Plaster cast and inner core. B. Outer shell. C. Finished dual durometer mock residual limbs.

Using a prosthetic vertical transfer jig to hold each of the molds, VytaFlex^™^ 20 urethane rubber (hardness shore 20A, Smooth-On, Reynolds Advanced Materials, Chicago, IL) was poured into the plaster cast before the inner core mold was lowered into the center to create the outer compliant shell (Fig 3A). The inner core mold was removed just prior to full curing (approximately 1 hour) leaving a central cavity (Fig 3B). Urethane adhesive (URE-BOND^™^ II, Smooth-On, Reynolds Advanced Materials, Chicago, IL) was applied to the inside of the compliant outer shell to promote adherence between the two urethane layers and a prosthetic pylon centered within the central cavity. VytaFlex^™^ 60 urethane rubber (hardness shore 60A, Smooth-On, Reynolds Advanced Materials, Chicago, IL) was poured into the central cavity and a 300 mm long stainless steel prosthetic pylon (Tube-Adapter, Long, 2R3, Diameter 30 mm, Ottobock, Louisville, KY) lowered into the liquid urethane rubber. The prosthetic pylon was pre-drilled with holes that had aluminum wire woven through them to create a mechanical lock with the urethane rubber of the inner core to minimize rotation. After curing for 48 hours, the plaster cast was carefully removed from the dual durometer mock residual limb using a cast saw. A total of 4 mock residual limbs were fabricated identically (Fig 3C).

### Test set-up

Mechanical testing was conducted using a hydraulic uniaxial material testing system (Instron, Norwood, MA). Mounting fixtures were designed using computer-aided design (CAD) software (SolidWorks Premium 2017 x64 Edition SP 2.0, Dassault Systèmes SolidWorks Corp., Waltham, MA) and fabricated from 50 mm^2^ aluminum square stock (6061 Aluminum, McMaster-Carr, Elmhurst, IL). Two mounting fixtures with male pyramid adaptors were attached to the Instron: one fixture was attached to the load cell (1000 lb (4448.22 N), MTS, Eden Prairie, MN) located in the top header of the Instron utilizing a threaded step stud (1"– 14 (2.54 cm—14); Thread 1 Type; Thread 2: 1/4"– 28 (0.635 cm—28), MTT Material Testing Technology, Wheeling, IL) and the other fixture to the moving piston at the bottom of the Instron.

An elevated vacuum attachment plate (EV-AP4-BK-CAUC, Prosthetic Design Inc., Dayton, OH) and manifold with air fitting (EV-MAN, Prosthetic Design Inc., Dayton, OH) were attached to the distal end of the socket. A 3 mm liner (Evolution Standard Liner, EOC-4, Össur Americas, Foothill Ranch, CA) and one nylon stocking were donned over the mock residual limb, which was then placed inside the socket. A sealing sleeve (Harmony Sleeve, 454A7 = K, Ottobock North America, Austin, TX) was rolled onto the exterior of the socket and sealed to the proximal portion of the liner above the proximal socket trim lines. To prevent air from leaking, electrical tape was used to seal the distal end of the sleeve to the socket. Two different suspension conditions were tested: passive suction and active vacuum. Passive suction was created by attaching a small piece of tubing and one-way valve to the manifold at the distal end of the socket. Active vacuum was created with an electronic vacuum pump (LimbLogic^™^, WillowWood, Mt. Sterling, OH) connected via tubing to the manifold at the distal end of the socket. Vacuum was set to 20 inHg (67.73 kPa) during testing.

### Testing procedure

The mock residual limb and socket were positioned in the test set-up and aligned at the top and bottom such that no offset moments were present. Compression of 50 N was applied to ensure that the mock residual limb was seated distally inside the socket and this position secured with screws at the top and bottom of the pyramid attachments. Tuning of the Instron was performed according to manufacturer instructions [38] for both displacement and load control for each socket and mock residual limb before data collection.

**Fig 4:**
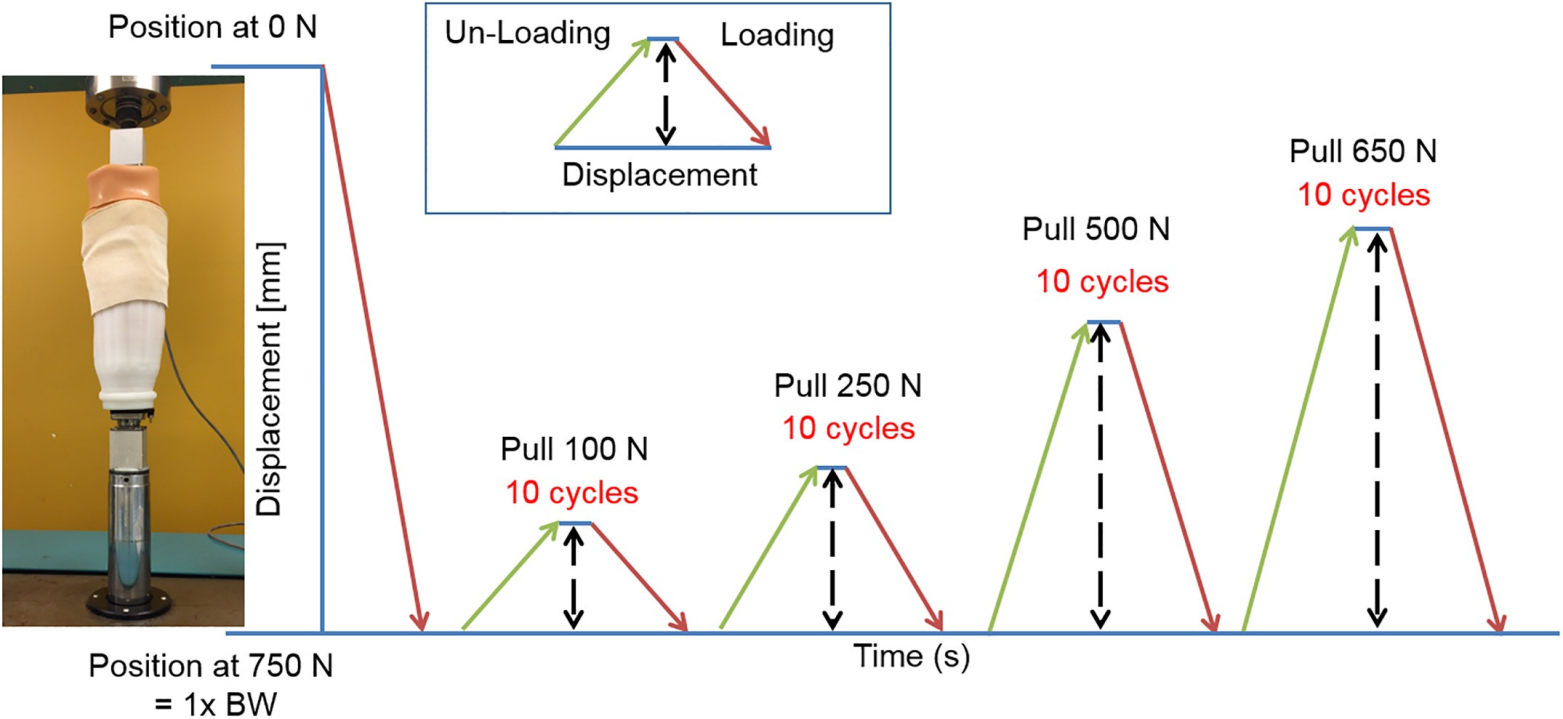
Protocol used for one testing trial: After compressing to 750 N, the socket was subjected to six trials of ten cycles at four increasing distraction force levels.

During testing, compression of 750 N was applied to the mock residual limb and socket to mimic the single-limb stance load exerted by a prosthesis user with body weight of approximately 75 kg. This load limit was determined based on pilot testing wherein the mock residual limb began to show visible surface damage at 1000 N of cyclic testing. Each socket was subjected to six trials consisting of ten cycles at each of four distraction force levels in the following order: 100 N, 250 N, 500 N and 650 N. We used an internal linear variable differential transformer (LVDT), which is part of the Instron, to measure crosshead movement and hence record socket displacement. As an example, during testing at the first distraction force level of 100 N, the mock residual limb inside the socket was cyclically unloaded to 650 N and then loaded back to 750 N ten times (i.e., a distraction force of 100 N pulled the socket away from the mock residual limb). Fig 4 depicts the protocol used for testing. Despite the initial 50 N of compression, additional settling of the mock residual limb inside the socket was observed during the first trial; therefore the first trial of data from each socket was discarded, leaving five trials for final analysis.

To assess repeatability of our testing procedure, we assessed the variation in measurements taken by a single person under the same conditions for three different specimens. We tested one rigid metal specimen on two separate days and two sockets (OSS and half-hemisphere LS) fit to a rigid plaster mock residual limb, each on a separate day. Additional details of the repeatability testing procedure are described in the S2 Appendix. We compared repeatability of the linear displacement data collected with the two sockets to linear displacement data collected with the metal specimen. Coefficient of variation for displacement measurements throughout the day were 0% for the metal specimen, 3.5% for OSS and 4.8% for half-hemisphere LS sockets. There was no relationship between displacement across trials with the metal specimen and time throughout each day (day 1, Pearson’s r = 0.0722 and day 2, Pearson’s r = -0.0369). By comparison, a moderate negative correlation (OSS socket, Pearson’s r = -0.4928) and a strong negative correlation (half-hemisphere LS socket, Pearson’s r = -0.8834) between measured displacements over time was observed throughout each day for the sockets. Since the same change was not observed when testing the metal specimen, we believe we can eliminate the possibility of overheating of the Instron from a day of non-stop testing as a contributing factor. It is more likely that changes seen over the course of the day when testing the sockets were due to the flow characteristics of the liner [39, 40] and its interaction with the textured socket. As a precaution against this, we avoided testing the same liner more than once per day. Since the mock residual limbs were made from urethane, which may also exhibit flow characteristics, we avoided testing the same mock residual limb more than once per day.

### Data analysis

Linear displacements, between the mock residual limb and socket were recorded, along with the corresponding forces, and analyzed using custom MATLAB (MathWorks, Natick, MA) codes. Statistical analyses were performed using SPSS version 25 (IBM, Armonk, NY) with linear displacement as the dependent variable having four distraction force levels (100 N, 250 N, 500 N and 650 N). We assessed the displacement of 16 different textures: 14 novel textures and two reference textures (smooth socket and OSS). Each texture was tested six times under two different conditions: passive suction suspension using a one-way valve (OV) and active vacuum suspension (VAC) using an electronic pump with the last five trials used for analysis. Therefore, there were three independent variables for all analyses: texture (a between socket measure), and suspension and distraction force level (both of which were within socket, repeated measures). While displacement was our dependent variable, it was measured for eight different conditions (4 distraction force levels x 2 suspension conditions). Sample size was n = 45, where nine cycles per each force level were assessed from five trials.

Given the number of independent and dependent variables, we conducted two types of statistical tests. First, we conducted a three-way mixed analysis of variance “between within within” (ANOVA BWW) to examine three-way interactions, two-way interactions, simple-simple main effects and simple comparisons, which was conducted between the suspension conditions for individual texture types at each distraction force level. Once we established that the ANOVA BWW was significant, we followed-up with Welch one-way ANOVAs to directly assess our hypotheses of whether there was a main effect of texture for each suspension condition and distraction force level.

During the initial ANOVA BWW analysis, the Shapiro–Wilk test indicated that displacement data were not normally distributed, hence we normalized displacement data using a two-step rank procedure [41]. Although homogeneity of variance was violated, our group sizes were equal; therefore we proceeded with ANOVA analyses as they are generally robust to violations in this assumption. The ANOVA BWW was conducted between the suspension conditions for individual texture types at each distraction force level (i.e., 2 suspension conditions x 4 force levels). A Bonferroni correction was applied within SPSS for the ANOVA BWW analysis, and critical alpha set at 0.05. Where the resulting p-values had more than 0.000 significant figures, we reported the results as p<0.0005 as recommended in the Laerd Statistics SPSS Statistics Guide [42].

Given that the main ANOVA BWW analysis confirmed significant interactions and main effects among all the independent variables, we conducted Welch one-way ANOVAs to assess whether there was a main effect of texture for each suspension condition and distraction force level. For this analysis, critical alpha was initially set at 0.05, then corrected to a Bonferroni-adjusted alpha level of 0.00625 (alpha = 0.05/8) to account for the multiple comparisons. For each Welch one-way ANOVA that was significant, we conducted Games-Howell post-hoc analyses for pairwise comparisons between each reference socket (smooth and OSS) and the other textures for each suspension condition and distraction force level with a Bonferroni-adjusted alpha level of 0.000208 (alpha = 0.00625/30). As indicated above, where the resulting p-values had more than 0.000 significant figures we stated the results as p<0.0005 [42].

## Results

### Displacement results

The range of longitudinal displacement of all sockets increased with increasing distraction force level and was greater for passive suction than active vacuum suspension at every distraction force level (See S4 Appendix for graphs):

- (OV: 0.215 to 0.369 mm and VAC: 0.193 to 0.313 mm at 100 N distraction force;
- OV: 0.666 to 1.029 mm and VAC: 0.509 to 0.848 mm at 250 N distraction force;
- OV: 1.708 to 2.487 mm and VAC: 1.244 to 1.964 mm at 500 N distraction force;
- OV: 2.519 to 3.738 mm and VAC: 1.715 to 3.144 mm at 650 N distraction force.

### Interactions and main effects

There was a statistically significant three-way interaction between suspension, force level and texture, F(23.548, 1105.185) = 166.138, p < 0.0005, partial η2 = .780, ε = .523. There was also a statistically significant (p < 0.0005) simple two-way interaction between suspension and force level for all texture types, and a statistically significant (p < 0.0005) simple-simple main effect of suspension on all measured displacements for all force levels. More detailed statistical results are summarized in the S3 Appendix.

### Comparing novel textures to reference sockets

Longitudinal displacements between textured and reference sockets were compared for both suspension conditions at each of the four force levels. Specifically:

- for 100 N distraction force (OV: Welch’s F(15, 263.100) = 659.742, p < 0.0005; VAC: Welch’s F(15, 264.762) = 2179.221, p < 0.0005);
- for 250 N distraction force (OV: Welch’s F(15, 263.118) = 609.635, p < 0.0005; VAC: Welch’s F(15, 265.131) = 1895.480, p < 0.0005);
- for 500 N distraction force (OV: Welch’s F(15, 265.430) = 817.331, p < 0.0005; VAC: Welch’s F(15, 265.114) = 2135.436, p < 0.0005); and
- for 650 N distraction force (OV: Welch’s F(15, 264.919) = 985.831, p < 0.0005; VAC: Welch’s F(15, 264.901) = 2668.341, p < 0.0005).

Results of the Games-Howell post-hoc analyses are summarized in Table 1. In general, novel texturing significantly reduced longitudinal displacement compared to the reference sockets, particularly the smooth socket.

**Table 1:** Mean and Standard Deviation (SD) for longitudinal displacement at each distraction force for both suspension conditions (OV: Passive suction with one-way valve; VAC: Active vacuum suspension at 20 inHg (67.73 kPa); mm: Millimeter; LS: Light and sparse; HD: Heavy and dense).

Socket Sample	100 N		250 N		500 N		650 N	
	OV [mm]	VAC [mm]	OV [mm]	VAC [mm]	OV [mm]	VAC [mm]	OV [mm]	VAC [mm]
Smooth Thermoformed	0.215 (0.015) ‡	0.236 (0.006) #	0.724 (0.045)	0.602 (0.011) #	2.113 (0.028) #	1.471 (0.024) #	3.223 (0.040) #	2.309 (0.051) #
Original Squirt-Shape (OSS)	0.252 (0.010) ¥	0.222 (0.003) †	0.666 (0.066)	0.577 (0.019) †	1.790 (0.057) †	1.308 (0.054) †	2.659 (0.124) †	1.715 (0.135) †
Horizontal Line LS	0.281 (0.003) ¥ #	0.212 (0.004) † ‡	0.802 (0.006) ¥ #	0.583 (0.023)	2.016 (0.030) † #	1.478 (0.024) #	2.925 (0.028) † #	2.135 (0.024) † #
Horizontal Rectangle LS	0.301 (0.014) ¥ #	0.245 (0.006) ¥ #	0.845 (0.034) ¥ #	0.657 (0.009) ¥ #	2.103 (0.066) #	1.538 (0.017) ¥ #	3.127 (0.117) † #	2.240 (0.050) † #
Half-hemisphere LS	0.249 (0.013) ¥	0.193 (0.011) † ‡	0.750 (0.031) #	0.509 (0.030) † ‡	1.874 (0.048) † #	1.244 (0.099) †	2.688 (0.109) †	1.930 (0.052) † #
Checkered LS	0.336 (0.008) ¥ #	0.283 (0.002) ¥ #	0.939 (0.022) ¥ #	0.764 (0.007) ¥ #	2.299 (0.038) ¥ #	1.778 (0.013) ¥ #	3.205 (0.066) #	2.691 (0.033) ¥ #
Hemisphere LS	0.302 (0.008) ¥ #	0.263 (0.004) ¥ #	0.832 (0.014) ¥ #	0.711 (0.005) ¥ #	1.892 (0.031) † #	1.637 (0.031) ¥ #	2.783 (0.052) † #	2.381 (0.028) ¥ #
Vertical Rectangle LS	0.293 (0.008) ¥ #	0.248 (0.006) ¥ #	0.830 (0.022) ¥ #	0.669 (0.010) ¥ #	1.991 (0.050) † #	1.560 (0.017) ¥ #	2.911 (0.038) † #	2.268 (0.054) #
Vertical Line LS	0.313 (0.006) ¥ #	0.236 (0.009) #	0.896 (0.010) ¥ #	0.684 (0.010) ¥ #	2.205 (0.032) ¥ #	1.654 (0.028) ¥ #	3.077 (0.078) † #	2.447 (0.017) ¥ #
Horizontal Line HD	0.338 (0.006) ¥ #	0.292 (0.003) ¥ #	0.948 (0.015) ¥ #	0.791 (0.010) ¥ #	2.336 (0.029) ¥ #	1,827 (0.016) ¥ #	3.426 (0.055) # ¥	2.787 (0.028) ¥ #
Horizontal Rectangle HD	0.369 (0.017) ¥ #	0.313 (0.013) ¥ #	1.029 (0.045) ¥ #	0.848 (0.037) ¥ #	2.487 (0.090) ¥ #	1.964 (0.125) ¥ #	3.738 (0.144) # ¥	2.902 (0.041) ¥ #
Half-hemisphere HD	0.273 (0.005) ¥ #	0.234 (0.007) #	0.724 (0.044)	0.626 (0.006) ¥ #	1.708 (0.107) †	1.403 (0.019) † #	2.518 (0.188) †	2.050 (0.028) † #
Checkered HD	0.309 (0.011) ¥ #	0.276 (0.002) ¥ #	0.854 (0.018) ¥ #	0.729 (0.009) ¥ #	2.194 (0.035) ¥ #	1.737 (0.011) ¥ #	3.341 (0.029) ¥ #	2.512 (0.019) ¥ #
Hemisphere HD	0.305 (0.011) ¥ #	0.262 (0.004) ¥ #	0.881 (0.018) ¥ #	0.693 (0.007) ¥ #	2.172 (0.043) ¥ #	1.611 (0.021) ¥ #	3.086 (0.066) † #	2.574 (0.060) ¥ #
Vertical Rectangle HD	0.267 (0.007) ¥ #	0.252 (0.003) ¥ #	0.752 (0.022) #	0.644 (0.009) ¥ #	1.984 (0.022) † #	1.694 (0.016) ¥ #	3.086 (0.045) † #	2.622 (0.031) ¥ #
Vertical Line HD	0.250 (0.011) ¥	0.269 (0.002) ¥ #	0.693 (0.058)	0.741 (0.006) ¥ #	2.070 (0.025) † #	1.959 (0.054) ¥ #	3.503 (0.048) ¥ #	3.143 (0.158) ¥ #

^†^ Significantly smaller displacement than smooth socket, p < 0.0005.

^‡^ Significantly smaller displacement than OSS socket, p < 0.0005.

^¥^ Significantly larger displacement than smooth socket, p < 0.0005.

^#^ Significantly larger displacement than OSS socket, p < 0.0005.

### Comparing passive suction and active vacuum

Across all force levels, smaller displacements were observed with passive suction for 10 of 14 novel textured sockets when compared to the smooth socket (indicated by † in Table 1). When active vacuum was applied, fewer novel textured sockets reduced displacements compared to the two reference sockets (4 of 14 compared to the smooth socket, indicated by † in Table 1, and 2 of 14 compared to the OSS socket, indicated by ‡ in Table 1).

For the smallest distraction force of 100 N with passive suction, all the novel textured sockets and OSS socket had significantly larger displacements (p < 0.0005) than the smooth socket (indicated by ¥ in Table 1) and 12 of 14 novel textured sockets had significantly larger displacements (p < 0.0005) than the OSS socket (indicated by # in Table 1). With active vacuum, Half-Hemisphere LS, Horizontal Line LS, and OSS sockets had significantly smaller displacements (p < 0.0005) than the smooth socket (indicated by † in Table 1). Half-Hemisphere LS and Horizontal Line LS textures had significantly smaller displacements (p < 0.0005) than the OSS socket (indicated by ‡ in Table 1). Additionally, 10 of 14 and 12 of 14 novel textured sockets had significantly larger displacements (p < 0.0005) than the smooth (indicated by ¥ in Table 1) and OSS (indicated by # in Table 1) sockets, respectively.

For the next distraction force of 250 N with passive suction, none of the novel textured sockets had significantly smaller displacements than the OSS socket. Twelve of 14 novel textured sockets had significantly larger displacements (p < 0.0005) than the OSS socket (indicated by # in Table 1) and 10 of 14 novel textured sockets had significantly larger displacements (p < 0.0005) than the smooth socket (indicated by ¥ in Table 1). With active vacuum, Half-Hemisphere LS and OSS sockets had significantly smaller displacements (p < 0.0005) than the smooth socket (indicated by † in Table 1). Only the Half-Hemisphere LS texture had significantly smaller displacements (p < 0.0005) than the OSS socket (indicated by ‡ in Table 1). Additionally, 12 of 14 novel textured sockets had significantly larger displacements (p < 0.0005) than the smooth (indicated by ¥ in Table 1) and OSS (indicated by # in Table 1) sockets, respectively.

For the next distraction force of 500 N with passive suction, none of the novel textured sockets had significantly smaller displacements than the OSS socket. However, 7 of 14 novel textured sockets had significantly smaller displacements (p < 0.0005) than the smooth socket (indicated by † in Table 1). Additionally, 13 of 14 and 6 of 14 novel textured sockets had significantly larger displacements (p < 0.0005) than the OSS (indicated by # in Table 1) and smooth (indicated by ¥ in Table 1) sockets, respectively. With active vacuum, Half-Hemisphere LS, Half-Hemisphere HD, and OSS sockets had significantly smaller displacements (p < 0.0005) than the smooth socket (indicated by † in Table 1). However, none of the novel textured sockets had significantly smaller displacements than the OSS socket. Additionally, 13 of 14 and 11 of 14 novel textured sockets had significantly larger displacements (p < 0.0005) than the OSS (indicated by # in Table 1) and smooth (indicated by ¥ in Table 1) sockets, respectively.

For the largest distraction force of 650 N with passive suction, none of the novel textured socket had significantly smaller displacement than the OSS socket. However, 9 of 14 novel textured sockets and OSS socket had significantly smaller displacements (p < 0.0005) than the smooth socket (indicated by † in Table 1). Additionally, 12 of 14 and 4 of 14 novel textured sockets had significantly larger displacements (p < 0.0005) than the OSS (indicated by # in Table 1) and smooth (indicated by ¥ in Table 1) sockets, respectively. With active vacuum, Half-Hemisphere LS, Half-Hemisphere HD, Horizontal Line LS, Horizontal Rectangle LS, and OSS sockets had significantly smaller displacements (p < 0.0005) than the smooth socket (indicated by † in Table 1). However, none of the novel textured sockets had significantly smaller displacements (p < 0.0005) than the OSS socket. Additionally, 14 of 14 and 9 of 14 novel textured sockets had significantly larger displacements (p < 0.0005) than the OSS (indicated by # in Table 1) and smooth (indicated by ¥ in Table 1) sockets, respectively.

### Comparing type of novel texturing

**Fig 5:**
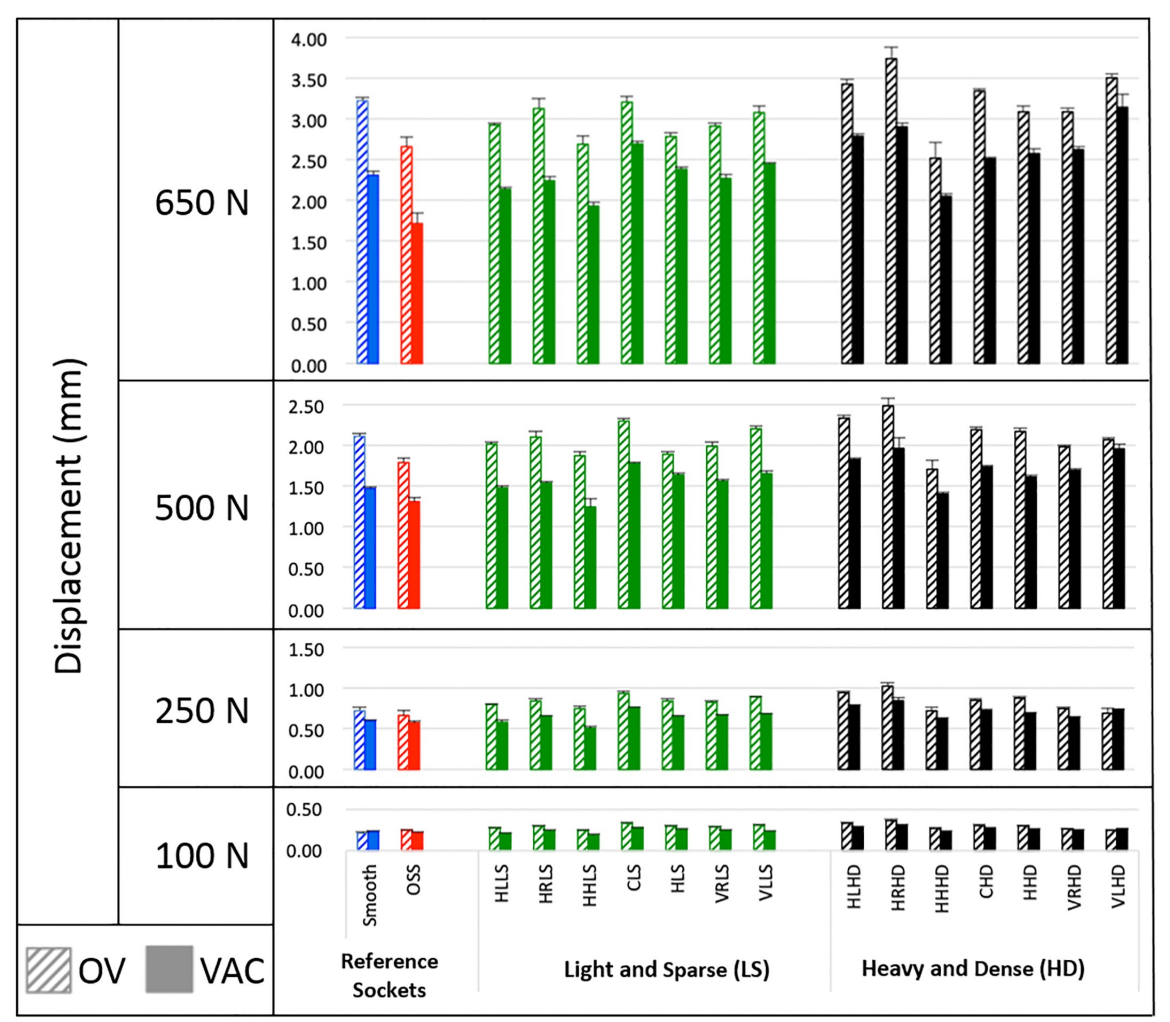
Longitudinal displacement for passive suction suspension with one-way valve (OV) and active vacuum with pump at 20 inHg (67.73 kPa) (VAC). Textures are organized from left to right from those with a primarily horizontal orientation of texture pattern to those with a primarily vertical orientation of texture pattern. Also, “heavy and dense” (HD) patterns are grouped to the right of “light and sparse” (LS) patterns.

Fig 5 illustrates measured displacements for the reference sockets compared to the novel textured sockets for each of the four force levels (100 N, 250 N, 500 N and 650 N) including the effect of suspension system on the measured displacement. Smaller displacement in the longitudinal direction was observed for the OSS socket when compared to the smooth socket. Smaller displacements in the longitudinal direction were observed under active vacuum for almost all of the novel textured sockets when compared to passive suction. There was no difference in displacement based on orientation of texture patterns from horizontal to vertical. Larger displacements were observed for more aggressive texture patterns as compared to more subtle texture patterns.

## Discussion

The aim of this study was to investigate longitudinal displacement of sockets with different types of textures under two suspension conditions: passive suction and active vacuum. Our hypothesis that novel textures would reduce longitudinal displacements compared to the smooth and OSS socket was partially supported. While some novel textures demonstrated smaller longitudinal displacements compared to the smooth socket, fewer novel textures demonstrated smaller longitudinal displacements compared to the OSS socket. Our hypothesis that active vacuum would provide smaller displacement for a given force in the longitudinal direction compared to passive suction was supported. Smaller longitudinal displacements were observed under active vacuum for almost all of the sockets compared to passive suction at all distraction force levels.

When considering whether more aggressive texture patterns and/or those with primarily horizontal pattern orientation resulted in less longitudinal displacement for a given force compared to more subtle texture patterns and/or those with primarily longitudinal pattern orientation, our results suggest that this was the case only for LS textures. LS sockets with texture patterns that had a primarily horizontal orientation decreased longitudinal displacement when compared to LS sockets with texture patterns that had a primarily vertical orientation. However, more aggressive HD texture patterns did not provide less displacement for a given force when compared to the more subtle LS texture patterns. An obvious limitation of this observation is that the ordering of texture patterns based on texture orientation was subjective. It would benefit future research if a more objective approach to classifying textures could be used. Additionally, differences in texture pattern were assessed in combination and as such it is not clear what might have resulted if we had made more systematic changes. Future research should assess these differences systematically, one difference at a time.

New test methods were developed to assess longitudinal socket suspension given that standardized tests are not available. The data suggest that the test protocol we developed has merit with regards to assessing longitudinal socket suspension. Firstly, changes in displacement scaled systematically with the distraction force levels applied such that the lowest distraction force resulted in the least displacement and the highest distraction force resulted in the most displacement. Secondly, these results were repeatable as there was little variability in displacement across the 10 cycles measured within each force level.

Our results are consistent with published studies reporting that vacuum suspension reduces pistoning compared to passive suction in human subjects [8, 9]. For example, Board et al. [8] used x-rays to measure socket pistoning while unweighted prosthetic limbs were statically distracted with loads of 44.5 N (simulating swing forces during walking) and 88.9 N (simulating swing forces during running). They reported liner displacements of 5±2 mm for passive suction and 1±1 mm for active vacuum. Klute et al. [9] asked subjects to weight and unweight their prosthesis while standing in place and limb pistoning was measured via a 12-camera motion analysis system. Limb pistoning of 1±3 mm was reported for active vacuum suspension. Displacement results in both these studies are similar to the displacement ranges observed in our study of 0.215 to 3.738 mm for passive suction and 0.193 to 3.144 mm for active vacuum.

The suspension conditions assessed in our study survived much higher distraction force levels than previously reported [27]. Gholizadeh et al. [27] used mechanical testing to evaluate four suspension systems during static tensile loading and observed suspension failure at distraction loads of 310 N for passive suction with a seal-in liner, 580 N for a pin locking liner, 351 N for a liner with a magnetic lock, and 490 N for a liner with a hook and loop Velcro strap, respectively. The investigators did not report whether they used compression preload to mimic body weight prior to applying the distraction load nor do they describe their mock residual limb construction. This lack of methodological detail makes it difficult to interpret why our results differ from those of Gholizadeh et al. [27].

The results of our study suggest that texturing may be used to improve socket longitudinal suspension and reduce pistoning compared to a smooth thermoformed socket. Ten out of fourteen textures outperformed the smooth thermoformed socket under passive suction and four out of fourteen textures outperformed the smooth thermoformed socket under active vacuum. However, under passive suction none of the novel textured sockets outperformed the OSS socket, while under active vacuum suspension, two novel textures (Half-Hemisphere LS and Horizontal Line LS) outperformed the OSS socket. Furthermore, longitudinal displacement was improved to a lesser degree under active vacuum compared to passive suction. Our results suggest that texturing of a socket with passive suspension improves the suspension forces that minimize longitudinal displacement so that it is comparable to a smooth socket with active vacuum. Given the additional cost of providing a pump for active vacuum suspension, and that it is relatively less expensive to print a socket using systems like the Squirt-Shape^™^ 3D Printer, texture may be considered the *“poor man’s vacuum”* (description attributed to Tracy Slemker, CPO, with permission).

A limitation of this study was that the mock residual limb was not as compliant as an anatomical residual limb given the need to ensure durability. An earlier iteration of the mock residual limb had a softer shell made of urethane with 10A shore hardness. Unfortunately, this softer shell delaminated from the hard inner core during pilot testing. The next iteration of the mock residual limb with an outer shell made of urethane with a shore hardness of 20A showed signs of damage when loaded to 1000 N, leading us to identify 750 N as the loading level that did not visibly damage the mock residual limb. While a softer outer shell may have provided even more realistic engagement with socket textures, it could not sustain the repetitive loading required during testing. Hence, our results may overestimate the displacements that might occur *in vivo* when adding texture to 3D printed sockets. Another limitation of the mock residual limb is that it does not change shape relative to the socket, so suspension is never compromised in the way it might be when the anatomical limb changes shape due to muscle contraction or limb orientation. Hence, our results may represent a best case scenario with respect to maintaining suspension.

Another limitation is that there are no existing standards for testing socket suspension, leading us to develop our own test set-up and protocol. Longitudinal testing was limited to uniaxial loading, which does not mimic the variety of loading directions and related moments a prosthetic socket would experience during activities of daily living. Static failure tests are needed to determine whether additional texturing of sockets compromises structural safety and human subjects testing is needed to determine whether additional texturing of sockets enhances suspension without causing the prosthesis user discomfort.

Finally, these results apply only to the particular texture patterns and dimensions 3D printed into rigid polypropylene copolymer sockets using the Squirt-Shape^™^ 3D Printer. It is possible that different texture patterns (e.g. texture having relatively large depth but lightly distributed) might perform better than the combinations tested in this study. It is also possible that applying texture in a different manner (e.g. to flexible inner sockets or liners) might improve suspension. These other approaches to applying texture into the prosthetic interface to reduce longitudinal displacement deserve further attention.

## Conclusions

Using newly developed mechanical testing protocols, it was demonstrated that texturing of polypropylene copolymer sockets fabricated using the Squirt-Shape^™^ 3D Printer significantly decreased longitudinal displacements compared to a smooth thermoformed polypropylene socket. Ten out of fourteen textures outperformed the smooth thermoformed socket under passive suction and four out of fourteen under active vacuum. However, under passive suction none of the novel textured sockets outperformed the OSS socket, while under active vacuum suspension, two novel textures outperformed the OSS socket. The effect of texture on longitudinal displacement was greater for passive suction than active vacuum suspension. While these tests attempt to mimic real-world socket conditions, further testing is needed to assess how textures might behave *in vivo*.

## Supporting information

S1 Appendix Texture patterns assessed in the study. (PDF)

S2 Appendix Repeatability testing procedure. (PDF)

S3 Appendix Statistical analysis results. (PDF)

S4 Appendix Longitudinal displacement graphs. (PDF)
