# Supplementary material for: Using mechanical testing to assess the effect of lower-limb prosthetic socket texturing on longitudinal suspension
Source: PLoS One. 2020 Aug 19;15(8):e0237841. doi: 10.1371/journal.pone.0237841 (PMC7437898; doi:10.1371/journal.pone.0237841)
Supplement: S1 Appendix — (PDF) [file pone.0237841.s001.pdf]

S1 Appendix. Dimensions of texture patterns assessed in the study (LS: light and sparse; HD: heavy and dense**).**

	Socket Sample	Width x Height [mm]	Horizontal x Vertical Spacing [mm]	Depth [mm]
Reference Sockets	Original Squirt-Shape (OSS)	0.75 (height only)	N/A	1.2
	Smooth Thermoformed	N/A	N/A	N/A
Novel Textured Samples	Horizontal Line LS	0.75 x 3.6	11 (vertical only)	1
	Horizontal Line HD	0.75 x 3.6	4 (vertical only)	2
	Horizontal Rectangle LS	20 x 4.8	60 x 15	1
	Horizontal Rectangle HD	20 x 4.8	10 x 2	3
	Half-hemisphere LS	10 x 6	20 x 20	1
	Half-hemisphere HD	10 x 6	6 x 6	3
	Checkered LS	10 x 1.2	60 x 15	1
	Checkered HD	10 x 1.2	10 x 3	3
	Hemisphere LS	10 x 10.8	20 x 20	1
	Hemisphere HD	10 x 10.8	6 x 6	3
	Vertical Rectangle LS	4 x 20.4	60 x 15	1
	Vertical Rectangle HD	4 x 20.4	10 x 2	3
	Vertical Line LS	9 (width only)	45 (horizontal only)	0.5
	Vertical Line HD	9 (width only)	18 (horizontal only)	1
