# Supplementary material for: Using mechanical testing to assess the effect of lower-limb prosthetic socket texturing on longitudinal suspension
Source: PLoS One. 2020 Aug 19;15(8):e0237841. doi: 10.1371/journal.pone.0237841 (PMC7437898; doi:10.1371/journal.pone.0237841)

S2 Appendix: Repeatability testing procedure.

Fig 1. Test protocol with metal specimen to assess repeatability at 650 N distraction force.

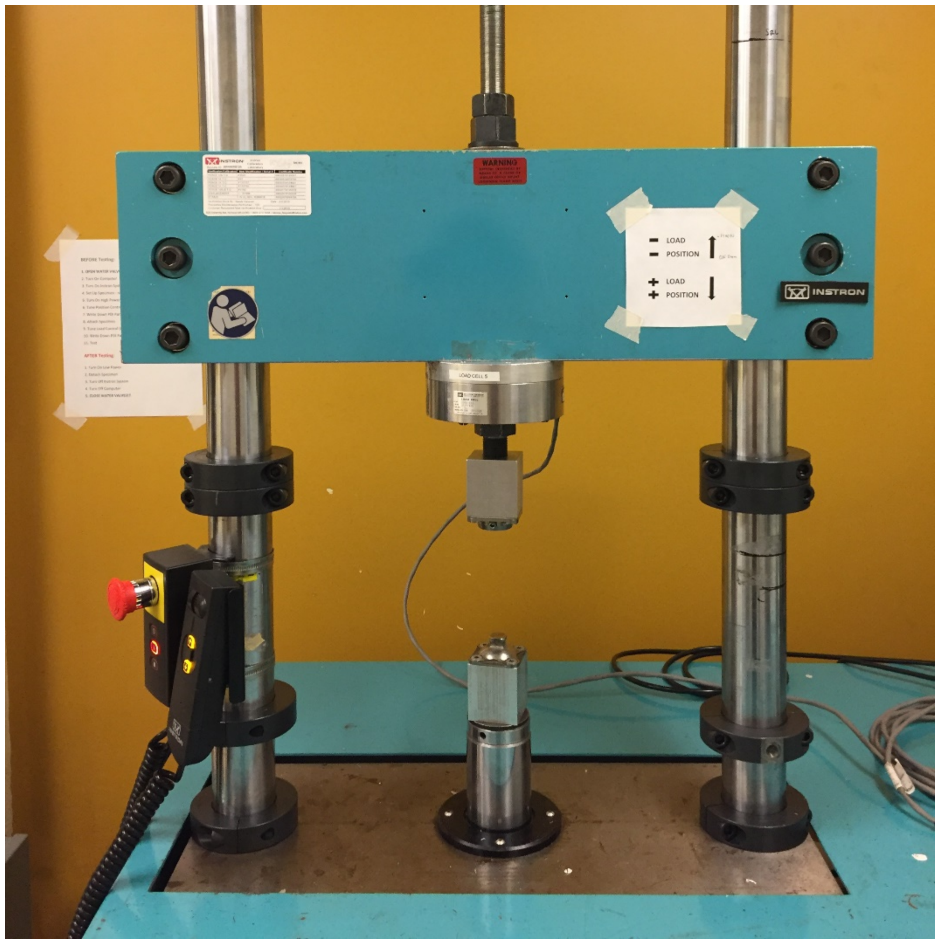

Metal specimen testing set-up.

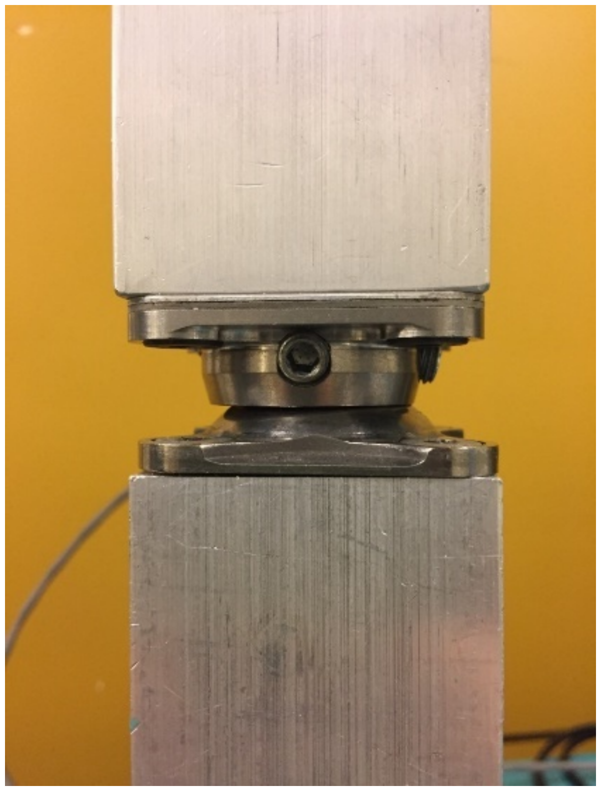

Metal specimen in full compression.

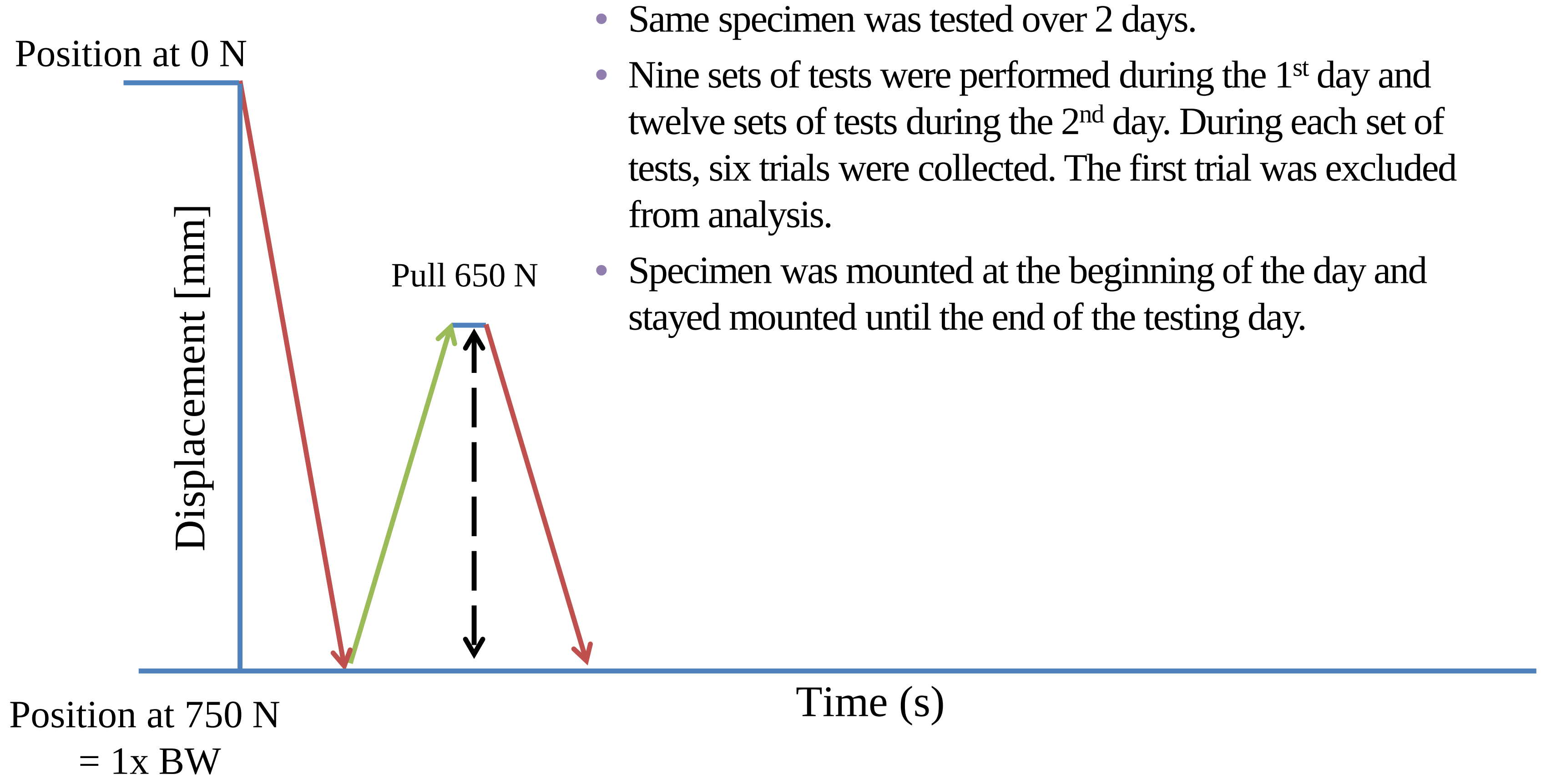

- Same specimen was tested over 2 days.
- Nine sets of tests were performed during the 1st day and twelve sets of tests during the 2nd day. During each set of tests, six trials were collected. The first trial was excluded from analysis.
- Specimen was mounted at the beginning of the day and stayed mounted until the end of the testing day.

Repeatability testing procedure - continued

Fig 2. Test protocol with two sockets to test repeatability at 650 N distraction force. (LS: light and sparse).

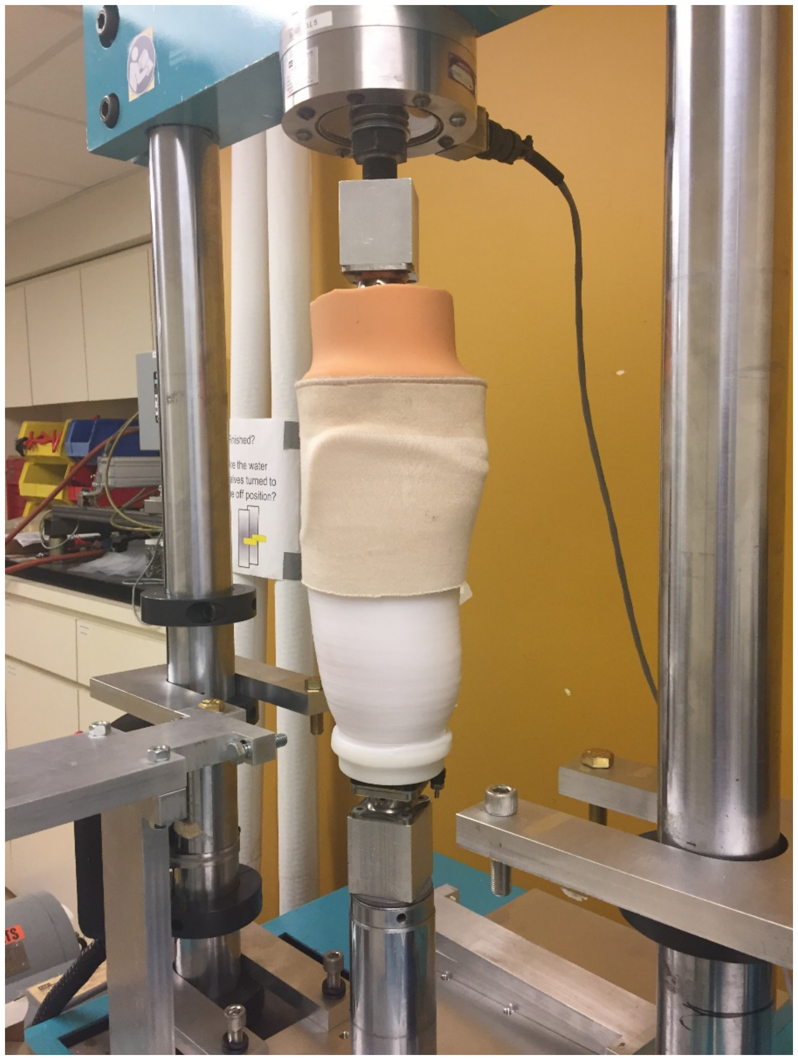

Original Squirt-Shape socket testing set-up.

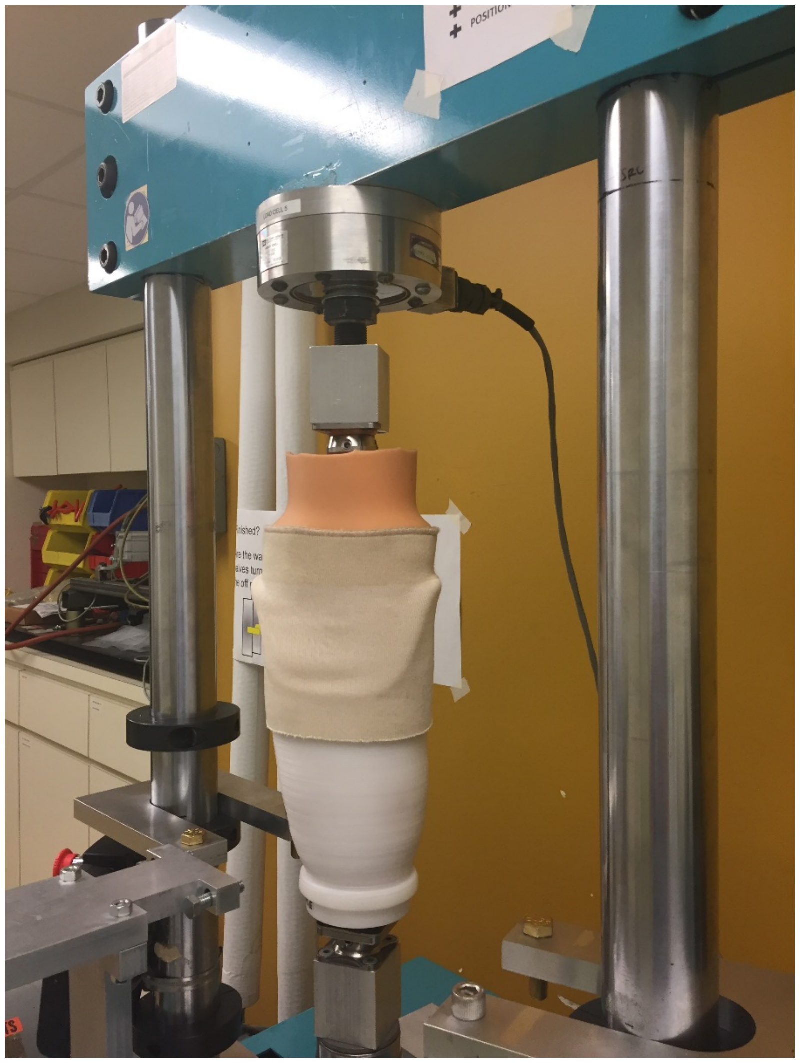

Half-Hemisphere LS socket testing set-up.

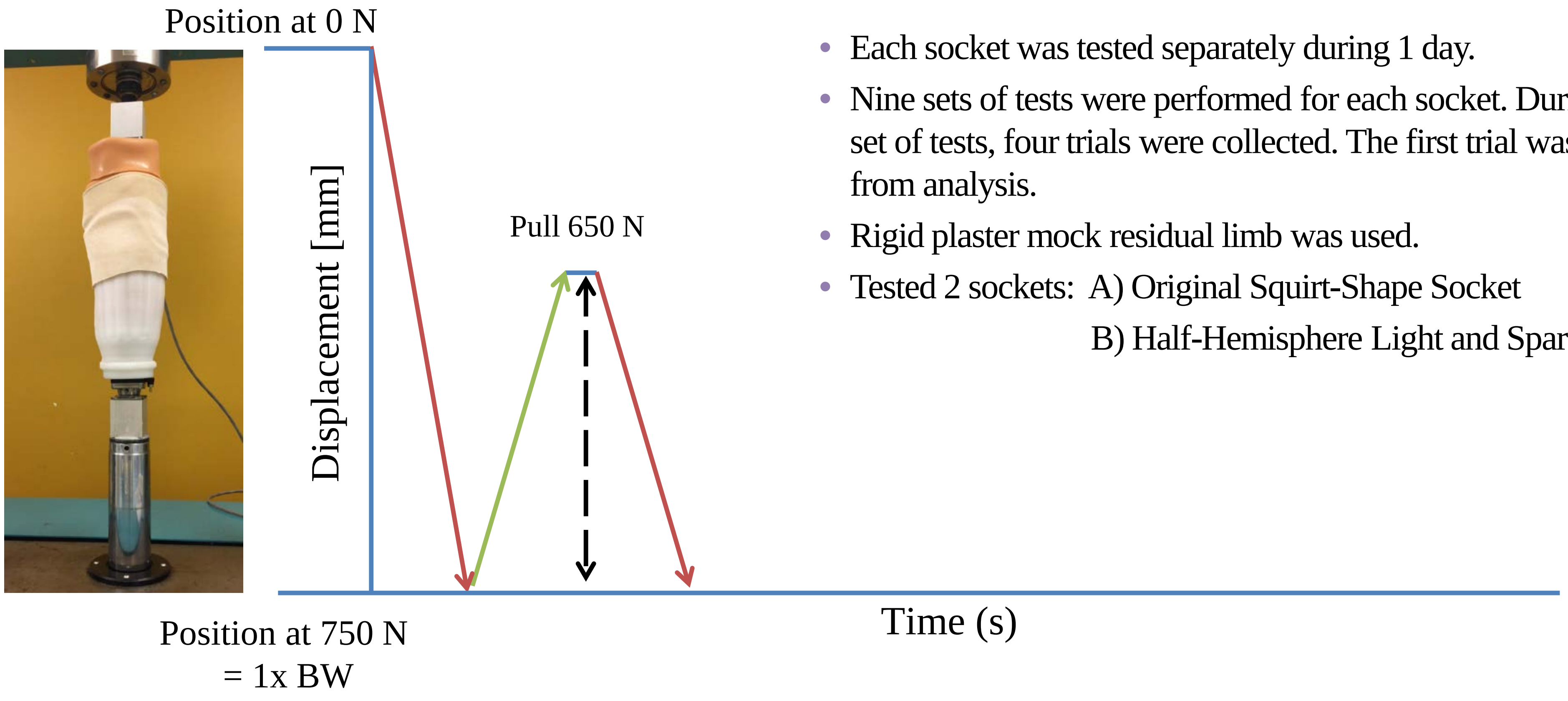

- Each socket was tested separately during 1 day.
- Nine sets of tests were performed for each socket. During each set of tests, four trials were collected. The first trial was excluded from analysis.
- Rigid plaster mock residual limb was used.
- Tested 2 sockets: A) Original Squirt-Shape Socket
  B) Half-Hemisphere Light and Sparse Socket

Repeatability testing procedure - continued

Fig 3 Repeatability testing results for metal specimen at 650 N distraction force tested on two separate days.

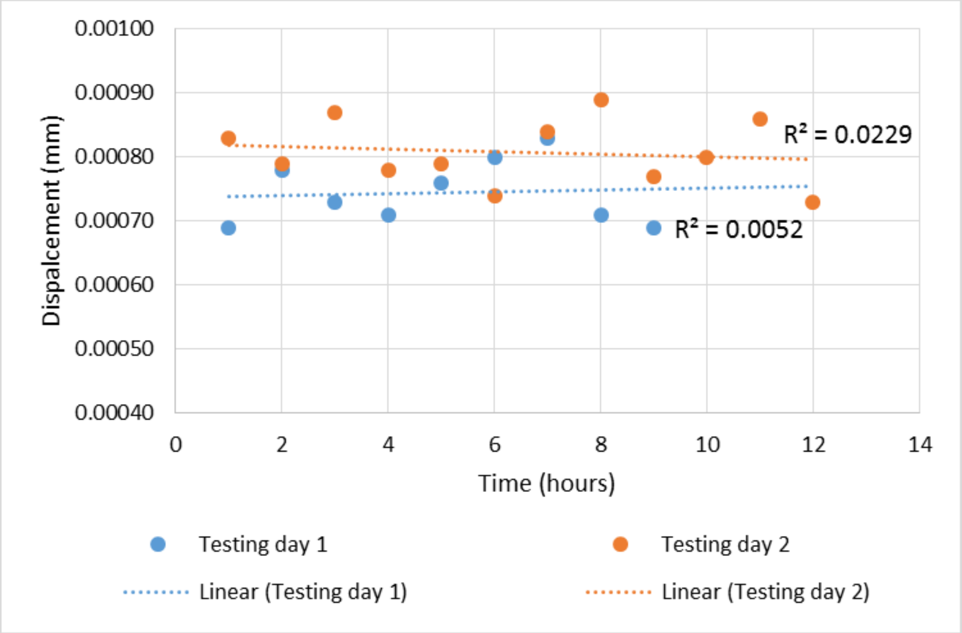

Fig 4. Repeatability testing results for sockets with plaster limb at 650 N distraction force tested on two separate days. (OSS: Original Squirt-Shape Socket, HH LS: Half-Hemisphere Light and Sparse).

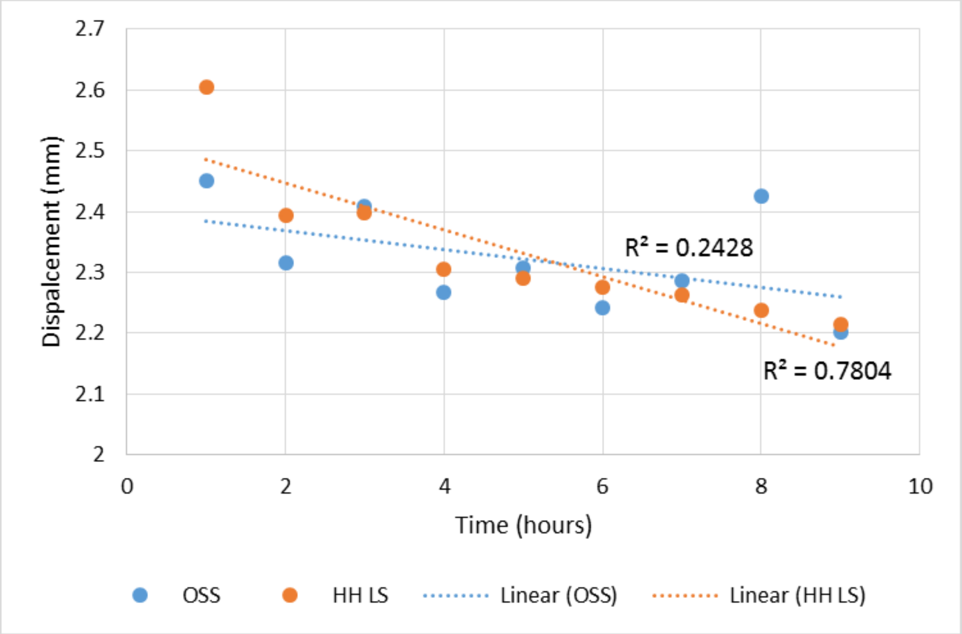

Supplement: S2 Appendix — (PDF) [file pone.0237841.s002.pdf]
