# Supplementary material for: Using mechanical testing to assess the effect of lower-limb prosthetic socket texturing on longitudinal suspension
Source: PLoS One. 2020 Aug 19;15(8):e0237841. doi: 10.1371/journal.pone.0237841 (PMC7437898; doi:10.1371/journal.pone.0237841)
Supplement: S3 Appendix — (PDF) [file pone.0237841.s003.pdf]

S3 Appendix: Statistical Analysis Results

Table 1. Three-way mixed ANOVA (BWW) procedure. Interaction between suspension condition and force level for longitudinal displacement (LS: light and sparse; HD: heavy and dense).

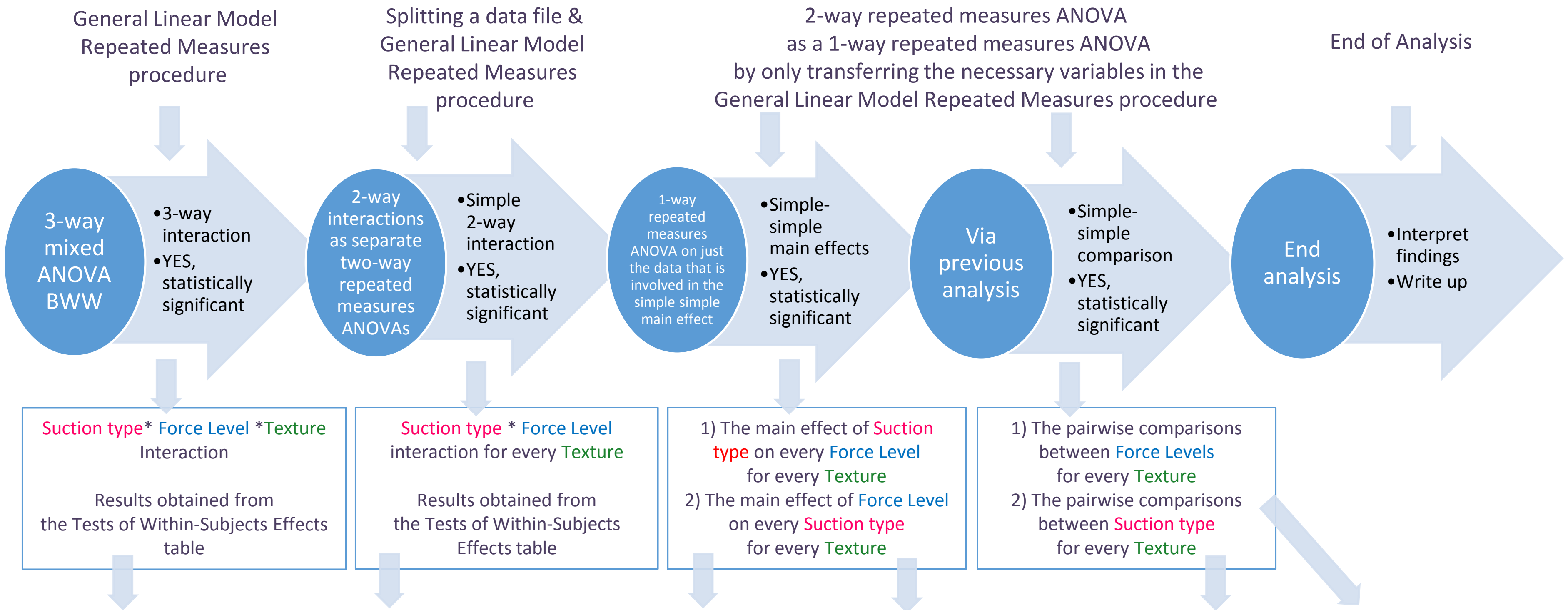

Yes, significant

Texture	2-way Interaction
C HD	Suction * Force Level
…	
Smooth	Suction * Force Level

Texture	Simple simple main effect
C HD	OV Suction effect on F100
	OV Suction effect on F250
	OV Suction effect on F500
	OV Suction effect on F650
	VACUUM Suction effect on F100
	VACUUM Suction effect on F250
	VACUUM Suction effect on F500
	VACUUM Suction effect on F650
…	
Smooth	

Texture	Simple simple main effect
C HD	F100 effect on OV Suction
	F100 effect on VACUUM Suction
	F250 effect on OV Suction
	F250 effect on VACUUM Suction
	F500 effect on OV Suction
	F500 effect on VACUUM Suction
	F650 effect on OV Suction
	F650 effect on VACUUM Suction
…	
Smooth	

C HD	F100 effect on:	F250
		F500
		F650
	F250 effect on:	F100
		F500
		F650
	F500 effect on:	F100
		F250
		F650
	F650 effect on:	F100
		F250
		F500
…		
Smooth		

C HD	OV Suction effect on:	VACUUM Suction
	VACUUM Suction effect on:	OV Suction
…		
Smooth		

S3 Appendix: Statistical Analysis Results

Table 2. **Three-way mixed ANOVA (BWW) two-way interaction results. Two-way interaction between suspension condition and force level for longitudinal displacement** (LS: light and sparse; HD: heavy and dense).

Socket Sample	Two-way interaction between suspension and force level
Original Squirt-Shape	F(1.226, 53.929) = 864.878, p < .0005
Smooth Thermoformed	F(1.798, 79.124) = 7002.047, p < .0005
Horizontal Line LS	F(1.856, 81.652) = 5928.795, p < .0005
Horizontal Rectangle LS	F(1.339, 58.905) = 5320.393, p < .0005
Half-Hemisphere LS	F(1.835, 80.736) = 1031.828, p < .0005
Checkered LS	F(1.237, 54.408) = 2836.796, p < .0005
Hemisphere LS	F(1.889, 83.099) = 3979.983, p < .0005
Vertical Rectangle LS	F(1.794, 78.925) = 4646.368, p < .0005
Vertical Line LS	F(1.216, 53.525) = 2644.928, p < .0005
Horizontal Line HD	F(1.298, 57.133) = 6957.062, p < .0005
Horizontal Rectangle HD	F(1.740, 76.547) = 1062.351, p < .0005
Half-Hemisphere HD	F(1.107, 48.702) = 252.401, p < .0005
Checkered HD	F(1.958, 86.164) = 20878.571, p < .0005
Hemisphere HD	F(1.191, 52.388) = 1531.699, p < .0005
Vertical Rectangle HD	F(1.627, 71.582) = 5136.838, p < .0005
Vertical Line HD	F(1.352, 59.471) = 215.641, p < .0005

Statistical Analysis Results - continued

Table 3. **Three-way mixed ANOVA (BWW) main effect results.** Main effect of suspension conditions on displacements for each force level (LS: light and sparse; HD: heavy and dense).

**Socket Sample**	**Simple-simple main effect of suspension on displacements for 100 N**	**Simple-simple main effect of suspension on displacements for 250 N**	**Simple-simple main effect of suspension on displacements for 500 N**	**Simple-simple main effect of suspension on displacements for 650 N**
Original Squirt-Shape	$F(1, 44) = 391.459, p < .0005$	$F(1, 44) = 82.626, p < .0005$	$F(1, 44) = 1619.825, p < .0005$	$F(1, 44) = 894.653, p < .0005$
Smooth Thermoformed	$F(1, 44) = 69.271, p < .0005$	$F(1, 44) = 344.855, p < .0005$	$F(1, 44) = 17911.810, p < .0005$	$F(1, 44) = 8279.648, p < .0005$
Horizontal Line LS	$F(1, 44) = 16193.067, p < .0005$	$F(1, 44) = 4233.511, p < .0005$	$F(1, 44) = 11001.238, p < .0005$	$F(1, 44) = 19621.220, p < .0005$
Horizontal Rectangle LS	$F(1, 44) = 1269.897, p < .0005$	$F(1, 44) = 2089.679, p < .0005$	$F(1, 44) = 5599.786, p < .0005$	$F(1, 44) = 5209.160, p < .0005$
Half-Hemisphere LS	$F(1, 44) = 623.821, p < .0005$	$F(1, 44) = 3378.468, p < .0005$	$F(1, 44) = 2431.675, p < .0005$	$F(1, 44) = 2634.541, p < .0005$
Checkered LS	$F(1, 44) = 2808.865, p < .0005$	$F(1, 44) = 5038.057, p < .0005$	$F(1, 44) = 12590.720, p < .0005$	$F(1, 44) = 2859.595, p < .0005$
Hemisphere LS	$F(1, 44) = 1858.081, p < .0005$	$F(1, 44) = 4898.796, p < .0005$	$F(1, 44) = 5180.818, p < .0005$	$F(1, 44) = 6839.626, p < .0005$
Vertical Rectangle LS	$F(1, 44) = 2078.552, p < .0005$	$F(1, 44) = 3648.830, p < .0005$	$F(1, 44) = 5179.855, p < .0005$	$F(1, 44) = 15775.968, p < .0005$
Vertical Line LS	$F(1, 44) = 2792.940, p < .0005$	$F(1, 44) = 17627.169, p < .0005$	$F(1, 44) = 21372.404, p < .0005$	$F(1, 44) = 3476.460, p < .0005$
Horizontal Line HD	$F(1, 44) = 2260.842, p < .0005$	$F(1, 44) = 7658.510, p < .0005$	$F(1, 44) = 22274.310, p < .0005$	$F(1, 44) = 7544.057, p < .0005$
Horizontal Rectangle HD	$F(1, 44) = 2783.974, p < .0005$	$F(1, 44) = 2129.049, p < .0005$	$F(1, 44) = 1974.770, p < .0005$	$F(1, 44) = 2282.643, p < .0005$
Half-Hemisphere HD	$F(1, 44) = 2641.898, p < .0005$	$F(1, 44) = 238.159, p < .0005$	$F(1, 44) = 457.787, p < .0005$	$F(1, 44) = 284.726, p < .0005$
Checkered HD	$F(1, 44) = 530.590, p < .0005$	$F(1, 44) = 2919.041, p < .0005$	$F(1, 44) = 7950.494, p < .0005$	$F(1, 44) = 22763.221, p < .0005$
Hemisphere HD	$F(1, 44) = 724.924, p < .0005$	$F(1,44) = 5244.811, p < .0005$	$F(1, 44) = 5869.926, p < .0005$	$F(1, 44) = 1295.197, p < .0005$
Vertical Rectangle HD	$F(1, 44) = 223.446, p < .0005$	$F(1, 44) = 1753.956, p < .0005$	$F(1, 44) = 9645.162, p < .0005$	$F(1, 44) = 5843.192, p < .0005$
Vertical Line HD	$F(1, 44) = 121.857, p < .0005$	$F(1, 44) = 36.855, p < .0005$	$F(1, 44) = 274.146, p < .0005$	$F(1, 44) = 230.774, p < .0005$

Statistical Analysis Results - continued

Table 4. **Three-way mixed ANOVA (BWW) pairwise comparison results for longitudinal displacement at distraction force of 100 N for both suspension conditions** (OV: passive suction with one-way valve; VAC: active vacuum suspension; LS: light and sparse; HD: heavy and dense).

Socket Sample	OV	VAC	Mean Difference (OV-VAC)	Std. Error	Sig.	95% Confidence Interval for Difference	
						Lower Bound	Upper Bound
Original Squirt-Shape	1	2	0.031	0.002	< 0.0005	0.027	0.034
Smooth Thermoformed	1	2	-0.020	0.002	< 0.0005	-0.025	-0.015
Horizontal Line LS	1	2	0.068	0.001	< 0.0005	0.067	0.070
Horizontal Rectangle LS	1	2	0.055	0.002	< 0.0005	0.052	0.058
Half-Hemisphere LS	1	2	0.056	0.002	< 0.0005	0.051	0.060
Checkered LS	1	2	0.053	0.001	< 0.0005	0.051	0.055
Hemisphere LS	1	2	0.039	0.001	< 0.0005	0.038	0.041
Vertical Rectangle LS	1	2	0.045	0.001	< 0.0005	0.043	0.047
Vertical Line LS	1	2	0.077	0.001	< 0.0005	0.074	0.080
Horizontal Line HD	1	2	0.045	0.001	< 0.0005	0.043	0.047
Horizontal Rectangle HD	1	2	0.057	0.001	< 0.0005	0.054	0.059
Half-Hemisphere HD	1	2	0.039	0.001	< 0.0005	0.037	0.040
Checkered HD	1	2	0.034	0.001	< 0.0005	0.031	0.037
Hemisphere HD	1	2	0.043	0.002	< 0.0005	0.040	0.046
Vertical Rectangle HD	1	2	0.016	0.001	< 0.0005	0.014	0.018
Vertical Line HD	1	2	-0.018	0.002	< 0.0005	-0.022	-0.015

Statistical Analysis Results - continued

Table 5. Three-way mixed ANOVA (BWW) pairwise comparison results for longitudinal displacement at distraction force of 250 N for both suspension conditions (OV: passive suction with one-way valve; VAC: active vacuum suspension; LS: light and sparse; HD: heavy and dense).

Socket Sample	OV	VAC	Mean Difference (OV-VAC)	Std. Error	Sig.	95% Confidence Interval for Difference	
						Lower Bound	Upper Bound
Original Squirt-Shape	1	2	0.088	0.010	< 0.0005	0.069	0.108
Smooth Thermoformed	1	2	0.122	0.007	< 0.0005	0.109	0.135
Horizontal Line LS	1	2	0.219	0.003	< 0.0005	0.212	0.226
Horizontal Rectangle LS	1	2	0.188	0.004	< 0.0005	0.180	0.197
Half-Hemisphere LS	1	2	0.241	0.004	< 0.0005	0.233	0.250
Checkered LS	1	2	0.176	0.002	< 0.0005	0.171	0.181
Hemisphere LS	1	2	0.121	0.002	< 0.0005	0.118	0.125
Vertical Rectangle LS	1	2	0.160	0.003	< 0.0005	0.155	0.166
Vertical Line LS	1	2	0.212	0.002	< 0.0005	0.208	0.215
Horizontal Line HD	1	2	0.158	0.002	< 0.0005	0.154	0.161
Horizontal Rectangle HD	1	2	0.181	0.004	< 0.0005	0.173	0.189
Half-Hemisphere HD	1	2	0.098	0.006	< 0.0005	0.085	0.111
Checkered HD	1	2	0.125	0.002	< 0.0005	0.120	0.130
Hemisphere HD	1	2	0.187	0.003	< 0.0005	0.182	0.193
Vertical Rectangle HD	1	2	0.108	0.003	< 0.0005	0.103	0.113
Vertical Line HD	1	2	-0.048	0.008	< 0.0005	-0.064	-0.032

Statistical Analysis Results - continued

Table 6. **Three-way mixed ANOVA (BWW) pairwise comparison results for longitudinal displacement at distraction force of 500 N for both suspension conditions** (OV: passive suction with one-way valve; VAC: active vacuum suspension; LS: light and sparse; HD: heavy and dense).

Socket Sample	OV	VAC	Mean Difference (OV-VAC)	Std. Error	Sig.	95% Confidence Interval for Difference	
						Lower Bound	Upper Bound
Original Squirt-Shape	1	2	0.482	0.012	< 0.0005	0.458	0.506
Smooth Thermoformed	1	2	0.642	0.005	< 0.0005	0.633	0.652
Horizontal Line LS	1	2	0.538	0.005	< 0.0005	0.528	0.549
Horizontal Rectangle LS	1	2	0.564	0.008	< 0.0005	0.549	0.580
Half-Hemisphere LS	1	2	0.630	0.013	< 0.0005	0.604	0.655
Checkered LS	1	2	0.521	0.005	< 0.0005	0.512	0.530
Hemisphere LS	1	2	0.254	0.004	< 0.0005	0.247	0.261
Vertical Rectangle LS	1	2	0.431	0.006	< 0.0005	0.419	0.443
Vertical Line LS	1	2	0.551	0.004	< 0.0005	0.543	0.559
Horizontal Line HD	1	2	0.509	0.003	< 0.0005	0.502	0.516
Horizontal Rectangle HD	1	2	0.523	0.012	< 0.0005	0.499	0.547
Half-Hemisphere HD	1	2	0.305	0.014	< 0.0005	0.277	0.334
Checkered HD	1	2	0.457	0.005	< 0.0005	0.447	0.467
Hemisphere HD	1	2	0.561	0.007	< 0.0005	0.546	0.576
Vertical Rectangle HD	1	2	0.290	0.003	< 0.0005	0.284	0.296
Vertical Line HD	1	2	0.110	0.007	< 0.0005	0.097	0.124

Table 7. **Three-way mixed ANOVA (BWW) pairwise comparison results for longitudinal displacement at distraction force of 650 N for both suspension conditions** (OV: passive suction with one-way valve; VAC: active vacuum suspension; LS: light and sparse; HD: heavy and dense).

Socket Sample	OV	VAC	Mean Difference (OV-VAC)	Std. Error	Sig.	95% Confidence Interval for Difference	
						Lower Bound	Upper Bound
Original Squirt-Shape	1	2	0.944	0.032	< 0.0005	0.880	1.007
Smooth Thermoformed	1	2	0.913	0.010	< 0.0005	0.893	0.934
Horizontal Line LS	1	2	0.790	0.006	< 0.0005	0.778	0.801
Horizontal Rectangle LS	1	2	0.887	0.012	< 0.0005	0.862	0.911
Half-Hemisphere LS	1	2	0.758	0.015	< 0.0005	0.728	0.788
Checkered LS	1	2	0.514	0.010	< 0.0005	0.495	0.533
Hemisphere LS	1	2	0.402	0.005	< 0.0005	0.392	0.412
Vertical Rectangle LS	1	2	0.643	0.005	< 0.0005	0.632	0.653
Vertical Line LS	1	2	0.631	0.011	< 0.0005	0.609	0.652
Horizontal Line HD	1	2	0.639	0.007	< 0.0005	0.624	0.653
Horizontal Rectangle HD	1	2	0.836	0.018	< 0.0005	0.801	0.872
Half-Hemisphere HD	1	2	0.468	0.028	< 0.0005	0.412	0.524
Checkered HD	1	2	0.829	0.005	< 0.0005	0.818	0.840
Hemisphere HD	1	2	0.511	0.014	< 0.0005	0.482	0.540
Vertical Rectangle HD	1	2	0.464	0.006	< 0.0005	0.452	0.476
Vertical Line HD	1	2	0.359	0.024	< 0.0005	0.312	0.407
