# Supplementary material for: Using mechanical testing to assess the effect of lower-limb prosthetic socket texturing on longitudinal suspension
Source: PLoS One. 2020 Aug 19;15(8):e0237841. doi: 10.1371/journal.pone.0237841 (PMC7437898; doi:10.1371/journal.pone.0237841)

# S4 Appendix: Longitudinal Displacement Graphs

**Figure 1**. **Mean longitudinal displacement for passive suction suspension with one-way valve (OV),** (LS: light and sparse; HD: heavy and dense).

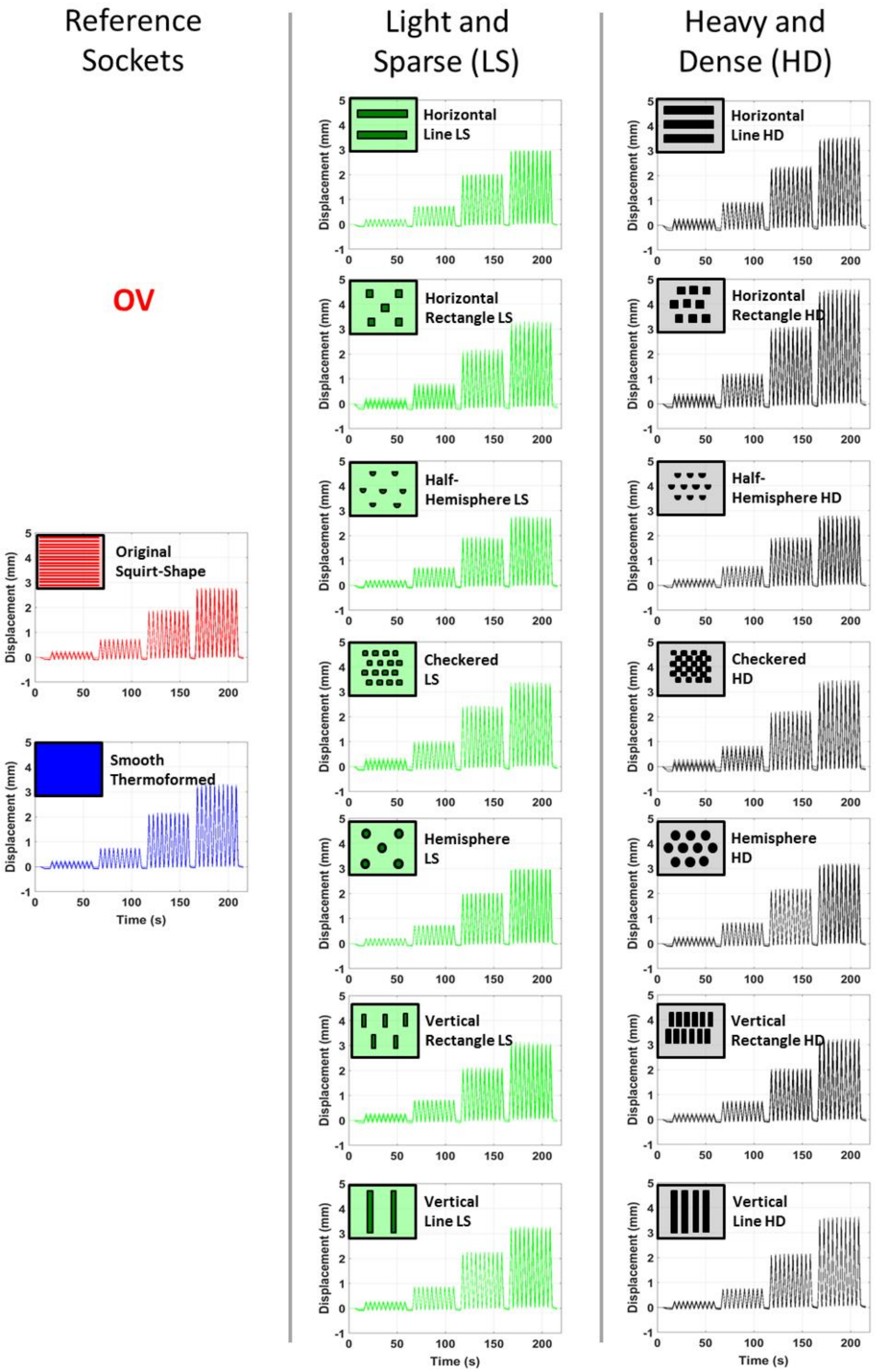

## Longitudinal Displacement Graphs - continued

**Figure 2**. **Mean longitudinal displacement for active vacuum with pump at 20 inHg (VAC),** (LS: light and sparse; HD: heavy and dense).

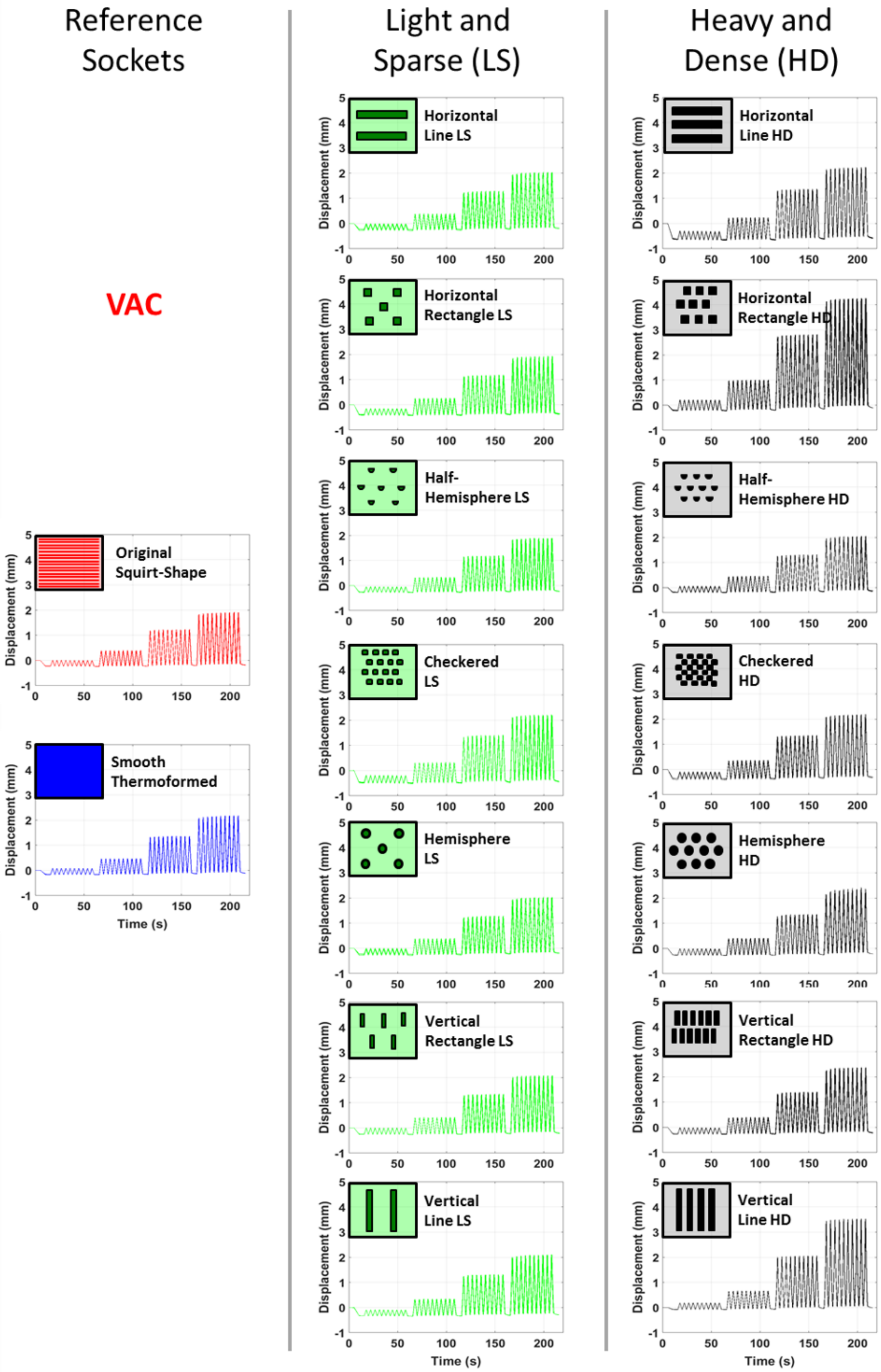

Supplement: S4 Appendix — (PDF) [file pone.0237841.s004.pdf]
